# Impact of headaches on university students in Durban, South Africa

**DOI:** 10.1186/s40064-016-3372-1

**Published:** 2016-09-29

**Authors:** Jyotika Basdav, Firoza Haffejee, T. Puckree

**Affiliations:** 1 Department of Chiropractic, Faculty of Health Sciences, Durban University of Technology, Gate 8, Steve Biko Road, Durban, South Africa; 2 Department of Basic Medical Sciences, Faculty of Health Sciences, Durban University of Technology, Gate 8, Steve Biko Road, Durban, South Africa; 3 Faculty of Health Sciences, Durban University of Technology, Gate 8, Steve Biko Road, Durban, South Africa

**Keywords:** Headaches, Primary, Epidemiology, Chiropractic, Impact, Students, University

## Abstract

**Background:**

Introspection into the factors that affect student success at higher education institutions has gained significant momentum in recent years. Teaching and learning has come under the spotlight with quality enhancement and teaching development funding focussing on student support, enhancing the student environment, and enhancing academics as teachers. Included in this are aspects that try to understand the student. An aspect that is not receiving attention is student health, specifically headaches which could impact student success. The aim of this study was to investigate the impact of primary headaches on student academic, family and social life at one higher education institution in South Africa.

**Method:**

Data was collected using a questionnaire based descriptive cross sectional survey. Multistage sampling using a ballot method allowed for sampling to obtain representation from across the institution. To achieve a 95 % confidence level, 384 students from across the university were invited to participate after informed consent. Data was analysed using Chi square tests at a probability of p < 0.05.

**Results:**

Majority of the participants were undergraduates and non-smokers. Half of the population suffered from primary headaches. Headache sufferers experienced limited concentration due to an increased headache intensity during tests and/or the examination period. This negatively impacted on studying which was aggravated by consumption of caffeinated energy drinks, coffee and chocolate resulting in a less effective study session. Activities of daily living and participation in social events which usually leads to relaxation were neglected. Personal and emotional well-being was also negatively affected. Altered sleeping patterns and absence of study breaks also led to headaches.

**Conclusion:**

Headaches were found to impact on the students study and sleep patterns, their attention levels during lectures and their social and emotional life. Headaches negatively impacted on some participants leading to reduced focus on academic, family, social or leisure activities. Intensity of headaches increased during tests and examinations which could impact their success at University.

## Background

Headaches affect almost half of the general population and about two thirds of the adolescent population, leading to a decreased quality of life (Jensen and Stovner 2008; Larsson and Fichtel 2014). Decreased productivity due to headaches has a negative impact on the economy due to absenteeism, loss of productivity and the resultant loss to the economy (Jensen and Stovner 2008; Hauch 1999; Radtke 2009). A study in Russia reports a 4 % decrease in productivity at work due to headaches (Ayzenberg et al. 2015). Days lost at work increased with headache severity (Wöber-Bingöl et al. 2014). Headaches result in functional disability, thereby impacting on the quality of life of the sufferers (Bussone et al. 2004). Those with chronic headaches are often unemployed or have significantly lower incomes than those who have episodic headaches or do not suffer from headaches at all (Zebenholzer et al. 2015).

Currently attention on student success in higher education has shifted focus to the factors that can be addressed to improve it (Education 2011). In South Africa, significant strides have been made to improve student support, the student learning environment and the quality of the academics as teachers through the Quality Enhancement Project (QEP) (Council of Higher Education (CHE) 2016) and Teaching Development Grants (TDG) (Department of Higher Education and Training 2014). All of these focus on improving student success. An aspect that still requires attention is student health, specifically headaches and their impact on student success.

Absenteeism from school due to headaches has affected students and their academic performance. A global study indicates that within a period of 4 weeks, a fifth of pupils with headaches lose at least one complete day from school; another fifth leave school early at least once, implying the loss of part of a day and almost half miss out on other school activities (Wöber-Bingöl et al. 2014). Furthermore, **s**tudents have difficulty in maintaining attention during lessons, and completing homework (Deleu et al. 2001; Smitherman et al. 2011; Curry and Green 2007; Menon and Kinnera 2013). In an American population, 51 % of the participants reported a decrease in school work and productivity by 50 % (Lipton et al. 2001). In a secondary school in Italy, 92 % of headache sufferers had difficulty in paying attention to lessons, participation in afternoon sport and completing homework tasks given to them (Tonini and Frediani 2012). Migraine sufferers missed twice as many days of classes and suffered reduced quality of life (Smitherman et al. 2011). Some headache sufferers reported missing as many as 3 days a week of lectures due to severe headaches which impacted their learning (Souza-e-Silva and Rocha-Filho 2011).

The impact of headaches has not been investigated in South African university students. The aim of this study was to determine the impact of primary headaches on students at a selected university in terms of academic performance, family and social lives.

## Methods

A descriptive cross sectional survey using a questionnaire allowed the collection of data on the impact of headaches on university students. The population consisted of students over the age of 18 years from a university of Technology. Multistage sampling allowed sampling from different strata namely campuses, faculties, programmes and levels of study. Students from two programmes in each faculty were randomly chosen using a ballot method. Thereafter, a further ballot method was used to select students from each level of study. From the total student population in the main campuses (22 303), using a confidence level of 95 % and a confidence interval of 5 %, a minimum required sample size of 384 students were invited to participate in the study. Students were drawn proportionally from each of the faculties in the following ratios: 25 % from Management Sciences (Marketing first year and Public Relations and Management first year), 24 % from Engineering and the Built Environment (Civil Engineering second year and Town and Regional Planning third year), 23 % from Accounting and Informatics (Bachelor of Technology in Information Technology and Financial Accounting first year), 11 % from Health Sciences (Emergency Medical Care and Rescue first year and Environmental Health second year), 10 % from Arts and Design (Photography first year and Translation and Interpreting Practice third year), and 8 % from Applied Sciences (Analytical Chemistry second year and Maritime Studies second year).

A questionnaire with open and closed ended questions, seeking information on primary headaches and their impact on the academic, social, emotional and family lives of the students at the University was adapted from that used by Prangley (2010) after the relevant permission to use these questions was obtained. The questionnaire was validated using an expert focus group and a pilot study. Both of these resulted in changes to the type of questions asked and more questions were added. The questionnaires were administered to the participants after obtaining ethical approval (Durban University of Technology Institutional Research Ethics Committee, IREC 002/15) to conduct the study, necessary gatekeeper permission and informed consent.

The International Classification of Headache Disorders (International Headache Society 2013) was used to classify primary headaches. Data was analysed using the Statistical Package for Social Sciences (SPSS) version 23.0. Chi square tests were use used to determine relationships between selected variables. Pearson Correlation coefficients were used to determine the correlations between different factors. Odds ratios were calculated to determine the likelihood of certain occurrences. A *p* value of less than 0.05 was considered statistically significant.

## Results

The response rate was 94 %. The mean age of the participants was 21 ± 3.08 years (range 18–25 years). The majority were 20–25 years old (52.9 %). Full demographic details are provided in Table 1. Half of the respondents suffered from primary headaches (50.2 %).

**Table 1:** Demographic profile of the participants

Age category	%
20–25	52.9
18–19	38.3
26–30	5.8
31–35	2.4
36–50	2.0
Female	60
Ethnic group	
Black	80.8
Indian	11.8
White	3.3
Coloured	3.1
Other	1.1
Undergraduate	98.1
Do not smoke	88.1

### Impact of headaches on academic life

The majority of participants (73.4 %) continued attending lectures while experiencing a headache. A large portion of the participants stated that headaches affected studying for tests and/or examinations (54 %). Majority reported that experiencing a headache limited their concentration (82.8 %; *p* < 0.001) and felt too tired to continue working (79.8 %; *p* < 0.001). Almost half of the participants (47.2 %) indicated that the headache was more intense than usual when studying for tests and exams *(p* < 0.001). Almost a third of participants that experienced a headache when studying, continued with the use of medication (31.4 %), and some (18 %) continued without the use of medication. However, a significant proportion (44.8 %) stopped studying due to the headache (*p* < 0.001).

Sleeping patterns were altered during tests and/or examination periods (67.6 %; *p* < 0.001). More than a third of the participants studied for long periods without taking regular breaks (39.6 %). A large number of participants consumed beverages such as caffeinated energy drinks, chocolate or coffee (63.1 %) to help sustain their concentration for a longer period of time. Consumption of these drinks during a headache made the study session less effective (*p* < 0.001). Lighting in the study area was adequate and did not affect studying (91.2 %; *p* < 0.001).

### Impact of headaches on daily activities

During headaches functionality with regard to daily tasks was reduced (71.3 %; *p* < 0.001) because it affected their energy levels (68.3 %; *p* < 0.001). Almost half of the participants (49.7 %; *p* = 0.92) required assistance in completing daily tasks such as household chores because they had to stop working to manage the headache (46.9 %; *p* = 0.21). Most of the respondents felt frustrated during a headache (64.2 %; *p* < 0.001) and felt that they were a burden on others at that point in time (36 %; *p* < 0.001). They also indicated that they were afraid of letting others down when experiencing a headache (44.1 %; *p* = 0.02). The headaches significantly affected the participants’ mood (82.8 %; *p* < 0.001), personal care (38.2 %; *p* < 0.001), lifting of objects (44.6 %; *p* = 0.04), reading (81.8 %; *p* < 0.001), concentration (88.9 %; *p* < 0.001) and performance in studies (65.1 %; *p* < 0.001).

### Impact on social life

Almost a third of the participants (30.7 %) neglected family, social or leisure activities due to their headaches. Almost half of the respondents (43.8 %) indicated that a headache sometimes stopped them from going out with family and/or friends. If a headache started during a social event, some participants either left early, requested medication, drank water or isolated themselves.

## Discussion

This study determined the impact of primary headaches on university students in South Africa. A novel finding is that the majority of students in this population continued attending lectures despite experiencing a headache. This is contradictory to other studies that reported significant absenteeism from school and college due to headaches (Smitherman et al. 2011; Menon and Kinnera 2013; Albers et al. 2015). However, there was a decreased ability to concentrate and this adversely affected studying for both tests and examinations. This was further exacerbated by altered sleeping patterns during headache episodes. Sleep disturbances have previously been reported to influence the frequency and duration of migraines (Waldie 2001; Al-Hashel et al. 2014). Despite the continued attendance at lectures, studying patterns were affected, with the majority unable to study. Many continued to study with the use of medication and some continued their studies without medication. Depending on the amount of time lost, this could have an adverse effect on test scores and pass rates particularly since those that continued studying reported a greater intensity of pain while studying. Furthermore, the frequency and intensity of headaches increased during the test and examination period. This could be linked to the stress that students experience during this time, since this was reported as a major trigger for headaches. The students in the current study consumed beverages such as energy and caffeinated drinks while studying, possibly as an attempt to stay awake for a longer period of time. However, Malinauskas et al. (2007) reported that 22 % of undergraduate students experience headaches as a side effect to energy drink consumption during study periods, with the consumption of three or more energy drinks causing a headache (Malinauskas et al. 2007). In the current study, the frequency of consumption of energy drinks during study periods was not determined but students need to be warned of the adverse effects of consuming large quantities of these drinks.

In addition, the occurrence of headaches leads to in a decrease in daily activities including household chores, as corroborated by other investigators (Radtke 2009; Menon and Kinnera 2013; Lipton et al. 2001; Amayo et al. 2002; Kernick and Reinhold 2002; Manandhar et al. 2015). Headaches affected the participants’ personal care and lifting of objects. This finding is consistent with that of other studies (Tonini and Frediani 2012). Students also report the fear of letting people down whilst experiencing headaches. Studies in an adult population have shown that in addition to the adverse effect on personal care and work, headaches also affect the spouse and children of the headache sufferer as children may be neglected and the spouse may also lose time from work (Steiner et al. 2014). Spouses of headache sufferers have also indicated that their relationship would have been better if the partner did not experience a headache (Lipton et al. 2001). The effects on social and family life were also reported by other investigators (Smitherman et al. 2011; Menon and Kinnera 2013; Lipton et al. 2001; D’Amico et al. 2003). Interference with communication, decreased the quality of time spent with the spouse and an increased number of arguments during a headache attack have also been previously reported (Lipton et al. 2001).

Our findings show that mood and emotional changes occur in students when they suffer from headaches. This is consistent with the report that depression is common in chronic headache sufferers and that this in turn decreases the quality of life (Zebenholzer et al. 2016). Giffin et al. (2003) described premonitory symptoms which were intensified during a migraine episode. The premonitory symptoms included tiredness, difficulty in concentration, irritability and being emotional. It was also found that moods greatly affected the intensity of the headache during an attack (Martin et al. 1988).

Our finding that 31 % of the study population used medication to relieve headaches, is much lower than that of the Italian population where 84 % of the population have been reported to use symptomatic drugs for headaches (Allena et al. 2015). This difference can be attributed to our sample only including students, whereas the latter study was conducted on the general population. Other studies also report low use of analgesics in adolescent populations (Albers et al. 2015; Birru et al. 2015; Krogh et al. 2016).

A limitation of the study is that we did not measure the severity of headaches which would have influenced the amount of lost productive time as shown in the working population (Manandhar et al. 2015).

## Conclusion

Headaches amongst university students negatively impact on both their academic as well as social lives. This study is of particular relevance to the discussions on and implementation of strategies to improve success rates in university students.

## References

[CR1] AFT Higher Education (2011) American Federation of Teachers, afl-cio (AFT). http://www.aft.org/sites/default/files/studentsuccess0311.pdf. Accessed 29 May 2016

[CR2] Albers L, Straube A, Landgraf MN, Filippopulos F, Heinen F, von Kries R (2015). Migraine and tension type headache in adolescents at grammar school in Germany—burden of disease and health care utilization. J Headache Pain.

[CR3] Al-Hashel JY, Ahmed SF, Alroughani R, Goadsby PJ (2014). Migraine among medical students in Kuwait University. J Headache Pain.

[CR4] Allena M, Steiner TJ, Sances G, Carugno B, Balsamo F, Nappi G, Andrée C, Tassorelli C (2015). Impact of headache disorders in Italy and the public-health and policy implications: a population-based study within the Eurolight Project. J Headache Pain.

[CR5] Amayo EO, Jowi JO, Njeru EK (2002). Headache associated disability in medical students at the Kenyatta National Hospital, Nairobi. East Afr Med J.

[CR6] Ayzenberg I, Katsarava Z, Sborowski A, Obermann M, Chernysh M, Osipova V, Tabeeva G, Steiner TJ (2015). Headache yesterday in Russia: its prevalence and impact, and their application in estimating the national burden attributable to headache disorders. J Headache Pain.

[CR7] Birru EM, Abay Z, Abdelwuha M (2015). Management of headache and associated factors among undergraduate medicine and health science students of University of Gondar, North West Ethiopia. J Headache Pain.

[CR8] Bussone G, Usai S, Grazzi L, Rigamonti A, Solari A, D’Amico D (2004). Disability and quality of life in different headaches: results from Italian studies. Neurol Sci.

[CR9] Council of Higher Education (CHE) (2016) Quality enhancement project (QEP). http://www.che.ac.za/focus_areas/quality_enhancement_project/overview. Accessed 29 May 2016

[CR10] Curry K, Green R (2007). Prevalence and management of headache in a university undergraduate population. J Am Acad Nurse Pract.

[CR11] D’Amico D, Usai S, Grazzi L, Rigamonti A, Solari A, Leone M, Bussone G (2003). Quality of life and disability in primary chronic daily headaches. Neurol Sci.

[CR12] Deleu D, Khan MA, Humaidan H, Mantheri Z, Al Hashami S (2001). Prevalence and clinical characteristics of headache in medical students in Oman. Headache.

[CR13] Department of Higher Education and Training (2014) Report of the Ministerial Committee for the Review of the Funding of Universities 2013. http://www.dhet.gov.za/SiteAssets/Latest%20News/Report%20of%20the%20Ministerial%20Committee%20for%20the%20Review%20of%20the%20Funding%20of%20Universities.pdf. Accessed 29 May 2016

[CR14] Giffin NJ, Ruggiero L, Lipton RB, Silberstein SD, Tvedskov JF, Olesen J, Altman J, Goadsby PJ, Macrae A (2003). Premonitory symptoms in migraine an electronic diary. Neurol.

[CR15] Hauch LE (1999) The impact of migraine headache. Dissertation, University of Nevada, Reno

[CR16] International Headache Society (2013). International classification of headache disorders. Cephalalgia.

[CR17] Jensen R, Stovner LJ (2008). Epidemiology and comorbidity of headache. Lancet Neurol.

[CR18] Kernick DP, Reinhold D (2002). The prevalence and treatement of headache sufficient to impact on the quality of life undergraduate students entering university. Curr Med Res Opin.

[CR19] Krogh A, Larsson B, Salvesen O, Linde M (2016). Assessment of headache characteristics in a general adolescent population: a comparison between retrospective interviews and prospective diary recordings. J Headache Pain.

[CR20] Larsson B, Fichtel A (2014). Headache prevalence and characteristics among adolescents in the general population: a comparison between retrospect questionnaire and prospective paper diary data. J Headache Pain.

[CR21] Lipton RB, Stewart WF, Diamond S, Diamond ML, Reed M (2001). Prevalence and burden of migraine in the United States: data from the American Migraine Study II. Headache.

[CR22] Malinauskas BM, Aeby VG, Overton RF, Carpenter-Aeby T, Barber-Heidal K (2007). A survey of energy drink consumption patterns among college students. Nutrit J.

[CR23] Manandhar K, Risal A, Linde M, Steiner TJ (2015). The burden of headache disorders in Nepal: estimates from a population-based survey. J Headache Pain.

[CR24] Martin PR, Nathan PR, Milech D, van Keppel M (1988). The relationship between headaches and mood. Behav Res Ther.

[CR25] Menon B, Kinnera N (2013). Prevalence and characteristics of migraine in medical students and its impact on their daily activities. Ann Indian Acad Neurol.

[CR26] Prangley J (2010) The primary headaches in allied health students at the Durban University of Technology (DUT), dissertation, Durban University of Technology. http://hdl.handle.net/10321/540. Accessed 29 May 2016

[CR27] Radtke A (2009). Prevalence and Burden of headache and migraine in Germany. Headache.

[CR28] Smitherman TA, McDermott MJ, Buchanan EM (2011). Negative impact of episodic migraine on a university population: quality of life, functional impairment, and comorbid psychiatric symptoms. Headache.

[CR29] Souza-e-Silva HR, Rocha-Filho PA (2011). Headaches and academic performance in university students: a cross-sectional study. Headache.

[CR30] Steiner TJ, Stovner LJ, Katsarava Z, Lainez JM, Lampl C, Lantéri-Minet M (2014). The impact of headache in Europe: principal results of the Eurolight project. J Headache Pain.

[CR31] Tonini MC, Frediani F (2012). Headache at high school: clinical characteristics and impact. Neurol Sci.

[CR32] Waldie KE (2001). Childhood headache, stress in adolescence, and primary headache in yound adulthood: a longitudinal cohort study. Headache.

[CR33] Wöber-Bingöl Ç, Wöber C, Uluduz D, Uygunoğlu U, Aslan TS, Kernmayer M, Zesch HE, Gerges NT, Wagner G, Siva A, Steiner TJ (2014). The global burden of headache in children and adolescents—developing a questionnaire and methodology for a global study. J Headache Pain.

[CR34] Zebenholzer K, Andree C, Lechner A, Broessner G, Lampl C, Luthringshausen G, Wuschitz A, Obmann SM, Berek K, Wöber C (2015). Prevalence, management and burden of episodic and chronic headaches—a crosssectional multicentre study in eight Austrian headache centres. J Headache Pain.

[CR35] Zebenholzer K, Lechner A, Broessner G, Lampl C, Luthringshausen G, Wuschitz A (2016). Impact of depression and anxiety on burden and management of episodic and chronic headaches—a cross-sectional multicentre study in eight Austrian headache centres. J Headache Pain.

